# Theta Activity During Encoding Interacts With NREM Sleep Oscillations to Predict Memory Generalization

**DOI:** 10.3389/fnhum.2022.821191

**Published:** 2022-05-09

**Authors:** Tamara Gibson, Zachariah R. Cross, Alex Chatburn

**Affiliations:** Cognitive Neuroscience Laboratory, Australian Research Centre for Interactive and Virtual Environments, University of South Australia, Adelaide, SA, Australia

**Keywords:** EEG, false memory, sleep spindles, theta, encoding, consolidation, generalisation

## Abstract

Relatively little is known regarding the interaction between encoding-related neural activity and sleep-based memory consolidation. One suggestion is that a function of encoding-related theta power may be to “tag” memories for subsequent processing during sleep. This study aimed to extend previous work on the relationships between sleep spindles, slow oscillation-spindle coupling, and task-related theta activity with a combined Deese-Roediger-McDermott (DRM) and nap paradigm. This allowed us to examine the influence of task- and sleep-related oscillatory activity on the recognition of both encoded list words and associative theme words. Thirty-three participants (29 females, mean age = 23.2 years) learned and recognised DRM lists separated by either a 2 h wake or sleep period. Mixed-effects modelling revealed the sleep condition endorsed more associative theme words and fewer list words in comparison to the wake group. Encoding-related theta power was also found to influence sleep spindle density, and this interaction was predictive of memory outcomes. The influence of encoding-related theta was specific to sleep spindle density, and did not appear to influence the strength of slow oscillation-spindle coupling as it relates to memory outcomes. The finding of interactions between wakeful and sleep oscillatory-related activity in promoting memory and learning has important implications for theoretical models of sleep-based memory consolidation.

## Introduction

One proposed function of episodic memory is to store information to allow accurate predictions about the environment. This is seen in particular through the established role of memory in influencing future behaviour, as has been shown in the relationship between episodic memory, long-term planning, and imagination (Hassabis et al., 2007; Szpunar et al., 2013). It should be noted, however, that consolidation (and the accompanying generation of schemata) must necessarily rely on both encoded material and encoding processes, although how encoding-related brain activity may influence consolidation processes has rarely been investigated, at least in terms of related EEG factors.

Emerging work suggests that encoding-related theta activity is associated with successful memory consolidation (Sederberg et al., 2003; Hasselmo and Stern, 2014), and that increased theta activity during learning predicts higher post-learning sleep spindle density (Heib et al., 2015). There is a body of evidence for a role of encoding theta on memory outcomes: successful recall of encoded information has been shown to improve as a function of pre-stimulus theta amplitude (Guderian et al., 2009; Cohen et al., 2015; Heib et al., 2015). Similarly, artificially inducing theta oscillations with transcranial slow oscillation stimulation during the encoding of information results in better recall of encoded information (Kirov et al., 2009), and theta power increases during encoding predict successful consolidation of episodic memory (Sederberg et al., 2007). Together, this work suggests that encoding-related neural activity influences memory consolidation, although the specific relationship between theta power and neurophysiological aspects of sleep-based memory consolidation has not been thoroughly investigated. One perspective is that theta power at encoding may serve a “tagging” function for subsequent memory processing during sleep (Heib et al., 2015).

To date, research has largely ignored the role of encoding-related electrophysiological activity as an influencer of sleep-based memory consolidation. This study aimed to investigate the influences of encoding-related theta activity on elements of sleep-based memory consolidation, such as sleep spindles and slow oscillation-spindle coupling on both learning and associations using the DRM paradigm. Theta power was estimated during the learning phase of the DRM, which was followed by a 2-h retention period of either typical wakefulness or a nap. It was predicted that increased theta power at encoding would result in improved recognition of DRM list words and worsened performance for associations (i.e., endorsement of critical lures). It was also hypothesised that greater sleep spindle density would result in increased veridical memories. We also sought to replicate the relationship between encoding-related theta activity and sleep spindles reported by Heib et al. (2015), and to extend this relationship to determine whether encoding-related theta interacts with slow oscillation-spindle coupling to influence memory outcomes.

## Materials and Methods

### Participants

Forty-three participants enquired, with 33 meeting the eligibility criteria. The final sample consisted of 35 participants between the ages of 18 and 33 (29 females, mean age = 23.2 years). Participants were randomly allocated into two groups, with 17 in the wake condition and 18 in the experimental nap condition. Sample size was determined through a G*Power calculation (Faul et al., 2007), which indicated that in order to obtain sufficient power (0.80) to detect a large effect size (0.80) at a significance level of 0.05, a sample of 30 participants was recommended (Diekelmann et al., 2008). All participants were healthy right-handed adults, who were not taking medication that could interfere with EEG, had not engaged in recreational drug use in the 6 months prior and were not diagnosed with a psychiatric or sleep disorder. The UniSA Human Research Ethics Committee granted ethics approval for the study, and all participants provided informed consent prior to participating. Participants received a $40 honorarium upon completion of the study.

### Screening Measures

The PSQI was used to measure self-reported sleep quality in the month leading up to participation to screen for poor sleep quality (Buysse et al., 1989; Gobin et al., 2015). Participants with a score of 5 or above (maximum possible score of 21) were excluded from participating. The Finders Handedness Survey (Flanders) was also completed by participants as a self-report measure of hand preference. Left handers were excluded from participating to mitigate handedness-related differences in the EEG (Nicholls et al., 2013).

### Electroencephalography

Electroencephalography data were collected using a BrainAmp BrainCap MR (Brain Products GmbH, Gilching, Germany), with 32-channel active DC Ag/AgCl electrodes. Electrodes were arranged according to the international 10-20 system (Gilmore, 1994). Bipolar electrooculogram (EOG) was also recorded, with electrodes placed 1 cm diagonally from the outer canthus of each eye. The EEG was sampled at 1000 Hz with impedances for electrodes kept below 10 kΩ. The online reference was located at FCz, and ground was AFz. EEG was continuously recorded during the DRM tasks, and during the sleep period.

### The Deese-Roediger-McDermott Paradigm

The DRM was used as a semantic memory association task (Roediger and McDermott, 1995), and was presented using *OpenSesame* (Mathôt et al., 2012). Eighteen themed word lists were used in order to test for memory for specific items as well as production of the overall gist of the word-lists. The DRM lists have been reported to elicit associative memories 77% (mean of every list) of the time (Stadler et al., 1999). Split-half measurements of the DRM display high internal consistency (*r* = 0.80; Stadler et al., 1999).

Each list consisted of 14 study words and 2 critical lure words (not presented during the learning phase), with the order of the words during the learning task arranged from most to least related to the lures (Roediger and McDermott, 1995). To improve the quality and accuracy of EEG analysis, the first words from each list were added as a critical lure, in keeping with Beato and Diez (2011).

**FIGURE 1:**
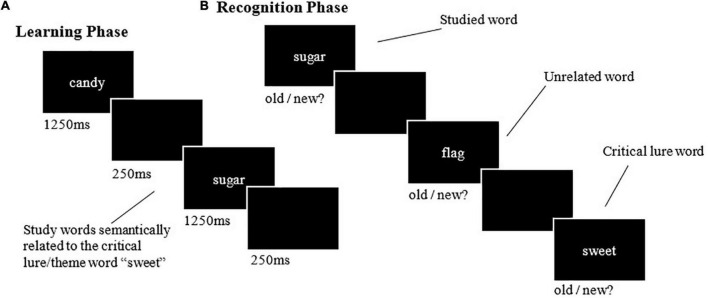
Example learning and recognition trials from the DRM paradigm. **(A)** The learning phase involved viewing lists of related study words. **(B)** The recognition phases involved immediate and delayed recognition, which involved the presentation of study words, their respective lures, and control words, to which participants made a self-paced old/new judgement.

The learning phase involved presenting all 14 words from the 18 experimental study lists in a serial visual presentation format. Each word was presented for 1250 ms. Each list word was followed by a blank screen for 250 ms, then a fixation cross for 500 ms. The learning phase took approximately 20 min. The recognition phases included the first, fifth, and eighth word from each presented list and the same words from unrelated control lists. Two critical lure words were also included for recognition from each presented list and the control lists (see Figures 1A,B for a schematic of the learning and recognition phases, respectively). Unlike the learning phase, the words were presented pseudo-randomly, to ensure no words from the same list were presented in succession. The presentation format and timing in the recognition phase were identical to that used in the learning phase. Participants responded to the presented words based on whether they thought the words were presented during the learning phase (old) or not (new). This was performed with a keyboard press, and these responses allowed for four outcomes to be calculated for veridical and association memories: (1) hit; (2) false alarm; (3) miss, and; (4) correct rejection [see Jano et al. (2021) for more details].

### Procedure

Participants arrived at approximately 10:30 a.m. at the UniSA Cognitive and Systems Neuroscience-Research Hub and completed screening tools (the Flanders, the PSQI, and a demographic questionnaire). Participants were asked to reduce their sleep the night prior to testing by 1 h to ensure participants experienced adequate sleep pressure for the afternoon nap, compliance of which was confirmed through self-report.

The EEG cap was then fitted, and participants were seated in a quiet testing room where the DRM paradigm was completed. EEG was recorded throughout the DRM task, and participants were instructed to do their best to commit list words to memory. Participants also undertook immediate recognition (IR) testing, in which a sub-list (three items) of each word list was presented to the participant, including the associative lures from each list. All conditions of stimuli presentation were consistent with the encoding phase. The same is true of the delayed recognition phase, in which the same content as in the immediate recognition and learning phases was presented in pseudo-randomised order.

**FIGURE 2:**
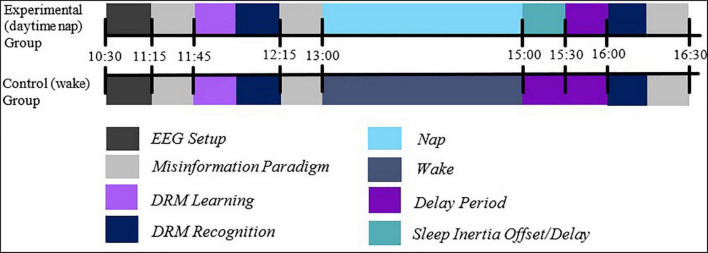
The experimental procedure. Participants were fitted with EEG caps and undertook learning of DRM lists, before being tested for immediate recognition and, following a sleep/wake retention interval, delayed recognition. Note that both IR and DR periods involved the presentation of associative theme words. Also note the presence of a misinformation task, which was unrelated to the present analysis.

Participants were randomly allocated to either the experimental (sleep), or to the control (wake) group. For the next 2 h, participants either napped or stayed awake and were given free time to do quiet study or browse the internet (control; see Figure 2). The 2-h period was chosen to allow enough time for one sleep cycle. To align with the post-lunch circadian dip (Monk, 2005), the nap occurred at approximately 1:00 p.m.

To compensate for sleep inertia (Achermann and Borbély, 1994), all participants were given a break of 1 h following the nap. Participants then completed the delayed recognition (DR) phase, which involved presenting the recognition task of the DRM, which was the same task and stimuli in the IR task, and participants’ EEG were again recorded. The recognition phase took approximately 15 min to complete.

## Data Analysis

### Deese-Roediger-McDermott Recognition Accuracy

Participant old/new responses were used to calculate response accuracy metrics (d’; McNicol, 2005) for both veridical and associative memories for both IR and DR tasks, as adapted from signal detection theory (McNicol, 2005). d’ was calculated using the package *Psycho* (Makowski, 2018) implemented in *R* version 3.6.1 (R Core Team, 2021). As per the methods used in Jano et al. (2021), d’ was calculated (Z-scored hits subtract Z-scored false alarms) for both list words and associative lures, in order to reflect our viewpoint that “false memory” measured in the DRM reflects associative processes, as opposed to memory errors.

### Sleep Scoring

An experienced technician followed the guidelines outlined by the American Academy of Sleep Medicine (AASM; Berry et al., 2012) to score participants’ sleep recordings in 30 s epochs. Information regarding total sleep time, sleep onset latency, and the percentage of time spent in sleep stages N1, N2, S3, and rapid eye movement (REM) sleep was acquired from this analysis.

### Sleep Spindle and Coupling Strength Detection

Sleep spindles were detected using the YASA toolbox implemented in MNE-Python (Vallat and Jajcay, 2020). The EEG signal was filtered between 12 and 16 Hz with a wide transition bandwidth of 1.5 Hz. The amplitude was calculated by applying a Hilbert transformation which was then smoothed with a 200 ms moving average. Candidate EEG phenomena which exceeded calculated thresholds were considered spindles, where the length of the spindle was determined by the length of time that absolute sigma power and covariance exceed their threshold. If the identified event was <0.3 s or >2.5 s, it was rejected. Slow oscillation-spindle coupling strength was also detected using the same toolbox, which was based on published algorithms (Helfrich et al., 2018). Slow oscillations were first extracted through continuous NREM EEG data and filtered using a digital phase-true FIR band-pass filter from 0.3 to 2 Hz with a 0.2 Hz transition band to detect zero crossing events that were between 0.3 and 1.5 s in length, and that met a 75–500 microvolt criterion. Slow oscillation-spindle coupling was detected using an event-locked cross frequency coupling metric. The normalised slow oscillation trough-locked data was filtered into the slow oscillation component (0.1–1.25 Hz) and extracted the instantaneous phase angle after applying a Hilbert transform. The same trials were then filtered between 12 and 16 Hz and then the instantaneous amplitude from the Hilbert transform was extracted. For each participant at channel Cz and epoch, the maximal sleep spindle amplitude and corresponding slow oscillation phase angle was calculated. The mean resultant vector length (mean vector length; coupling strength) across all NREM events was then determined using circular statistics implemented in the *pingouin* package (Vallat, 2018). A scale of 0–1 was used for mean vector length, with 1 indicating that each coupled spindle occurred at the same phase of the slow oscillation, and 0 indicating that each coupled spindle occurred at a different phase of the slow oscillation.

### Deese-Roediger-McDermott Time Frequency Analysis

Electroencephalography data were re-referenced to the average of linked mastoids and bandpass filtered from 1 to 40 Hz. Independent Component Analysis (*fastica)* was performed on EEG data to remove ocular and muscular artefacts. Data were further cleaned using the *autoreject* package (Jas et al., 2017). Spectral activity in the theta range (∼4–7 Hz) was estimated using a complex Morlet wavelet analysis implemented in MNE-Python (tfr_morlet). Participants’ theta frequency ranges were adjusted according to the golden mean algorithm (Klimesch, 2012). Theta power was estimated from −0 to 1000 ms post-word onset during the immediate and delayed DRM tasks. Baseline power values in the pre-stimulus (−200 to 0 ms) window were also measured and were included in the statistical models to control for pre-stimulus differences, as recommended by Alday (2019).

### Linear Mixed-Effects Models

Linear mixed effects models were constructed using the *lme4* package (Bates et al., 2015) in *R* (R Core Team, 2021) to assess the interactions between encoding-related theta power and consolidation-related sleep physiology on the generation of veridical and association memories. *P*-values were calculated through type II Wald tests as implemented in the *car* package (Fox, 2011). Visualisation of modelled effects were created through the *effects* (Fox et al., 2019) and *ggplot2* (Wickham et al., n.d.) packages. *Post hoc* testing, when appropriate, was performed with *emmeans* (Lenth, 2022), to obtain pairwise comparisons with Bonferroni-Holm correction applied for multiple comparisons.

To test hypothesis one (theta power increases at encoding would lead to an increase in memory for DRM list words and a decrease in endorsement of associative theme words), we aimed to predict d’ from task-evoked theta power, condition (sleep/wake) and memory type (veridical/association), while controlling for the effects of immediate recognition testing performance. Models also included mean pre-stimulus theta power as a scaled covariate in order to control for pre-stimulus activity (Alday, 2019), and by-channel random intercepts to account for topographical differences in theta power estimates. Electrode location across the scalp was also modelled by specifying sagittality (anterior/central/posterior) and laterality (left/midline/right) as fixed effects. By-participant random intercepts were also specified in each model. More formally, the LMM equation took the form:


$$d\,p\,r\,i\,m\,e_{i}=\beta_{0}+\beta_{1}\,t\,h\,e\,t\,a_{i}*\beta_{2}\,c\,o\,n\,d\,i\,t\,i\,o\,n_{i}*\beta_{3}\,t\,y\,p\,e_{i}*\beta_{4}\,l\,a\,t_{i}*\beta_{5}\,s\,a\,g+$$

$$\beta_{6}\,p\,r\,e\,s\,t\,i\,m_{i}+\beta_{7}\,I\,R_{i}+s\,u\,b\,j\,e\,c\,t_{0\,i}+c\,h\,a\,n\,n\,e\,l_{0\,i}+\varepsilon ,$$

To explore our second hypothesis (greater sleep spindle density would result in an increase in memory for DRM list words), as well as our research questions around the role of encoding-related theta power as a driver of both sleep spindle density as well as association of list words across the retention period, encoding-related theta power, sleep spindle density and memory type were used as predictors of d’ from the pre- to post-retention intervals in the sleep condition, once again, controlling for immediate recognition performance and encoding for topographical location of electrodes. This LMM equation took the form:


$$d\,p\,r\,i\,m\,e_{i}=\beta_{0}+\beta_{1}\,t\,h\,e\,t\,a_{i}*\beta_{2}\,d\,e\,n\,s\,i\,t\,y_{i}*\beta_{3}\,t\,y\,p\,e_{i}*\beta_{4}\,l\,a\,t_{i}*\beta_{5}\,s\,a\,g+$$

$$\beta_{6}\,p\,r\,e\,s\,t\,i\,m_{i}+\beta_{7}\,I\,R_{i}+s\,u\,b\,j\,e\,c\,t_{0\,i}+c\,h\,a\,n\,n\,e\,l_{0\,i}+\varepsilon ,$$

To test whether encoding-related theta power interacts with slow oscillation-spindle coupling strength to predict memory outcomes, we modelled d’ scores across retention period from theta power at encoding, memory type, slow oscillation-spindle coupling strength, from participants in the sleep condition.

## Results

d’ scores per condition are reported in Table 1. These results indicate adequate behavioural performance based on SDT measures. Sleep parameters from the experimental condition are reported in Table 2; broadly speaking, these values represent that participants obtained adequate sleep during the nap in order to provide valid data for subsequent analyses.

**TABLE 1:** Mean (SD) d’ scores across task, condition, and memory type.

Condition	Immediate recognition		Delayed recognition	
	List words	Theme words	List words	Theme words
Sleep	1.58 (0.64)	1.24 (0.37)	1.28 (0.64)	1.28 (0.50)
Wake	1.50 (0.43)	1.22 (0.43)	1.14 (0.44)	1.13 (0.55)

*SD, standard deviation.*

**TABLE 2:** Time spent asleep, sleep spindle, slow oscillation, and spindle\SO coupling strength as means, standard errors, and ranges.

Sleep parameters	Mean (SEM)	Range
TST	102.71 (2.00)	77.5–117.5
S1	17.50 (1.85)	2.50–43.00
S2	56.04 (3.37)	22.50–85.00
S3	9.58 (1.95)	0–36
REM	14.33 (1.58)	2.50–39
Spindle density	1.80 (2.49)	0.25–6.83
Slow Oscillation density	10.75 (0.53)	0.38–27.23
SO-spindle coupling strength (ndPAC)	0.22 (0.003)	0.19–0.26

*TST, total sleep time; S1, stage 1; S2, stage 2; S3, stage 3; REM, rapid eye movement; SO, slow oscillation.*

Behavioural results indicate a significant relationship between condition (sleep/wake) and type of memory (list word or associative lure) in determining memory for list words and associative lures [χ^2^(1) = 13.65, *p* < 0.001], such that associative lures were recognised with slightly greater accuracy than list words (see Figure 4A), consistent with classic findings that “false” memories are better remembered than veridical memories (Seamon et al., 2002). Given this, we turn our analyses to understanding the topographic distribution of theta power, with frontal theta in particular being noted to play a role in encoding and related mnemonic processes (Heib et al., 2015). To do this, LMM was used to predict differences in theta power, accounting for condition, memory type and including a fixed factor of pre-stimulus theta power, as well as random effects of subject and channel. This model took the form:


$$t\,h\,e\,t\,a_{i}=\beta_{0}+\beta_{1}\,c\,o\,n\,d\,i\,t\,i\,o\,n_{i}*\beta_{2}\,t\,y\,p\,e_{i}*\beta_{3}\,l\,a\,t_{i}*\beta_{4}\,s\,a\,g+\beta_{5}\,p\,r\,e\,s\,t\,i\,m_{i}+$$

$$s\,su\,b\,j\,e\,c\,t_{0\,i}+c\,h\,a\,n\,n\,e\,l_{0\,i}+\varepsilon .$$

**FIGURE 3:**
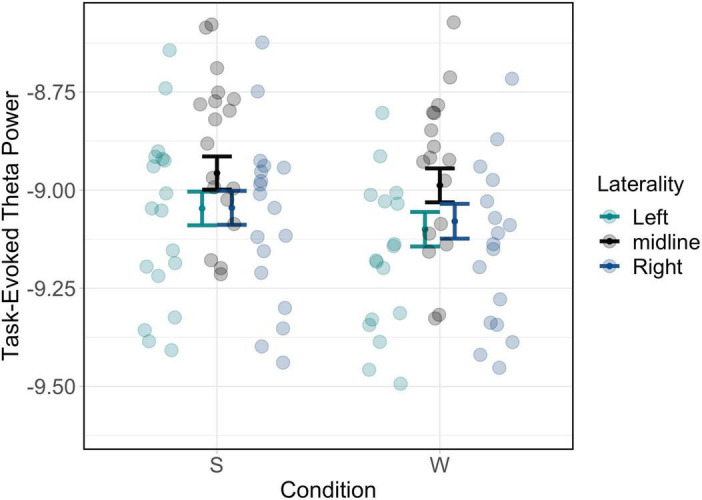
Theta power differences plotted by scalp region of interest for laterality. Significant effects of condition by laterality are driven by differences between midline vs. other locations.

This analysis indicated a significant laterality by condition interaction [χ^2^(2) = 15.35, *p* < 0.001]. *Post hoc* testing revealed that, for both wake and sleep conditions and true and false memory types, midline theta values were higher compared to both left and right lateralised locations (see Supplementary Materials); these values are plotted in Figure 3; tables of estimated marginal means are presented in appendices 1 and 2 in Supplementary Material. Subsequent analyses of the role of encoding-related theta power therefore focussed on midline electrodes.

**FIGURE 4:**
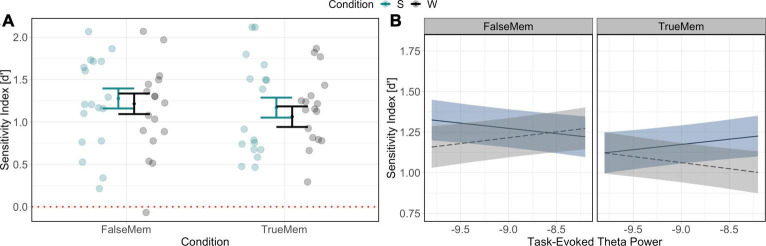
Behavioural **(A)** and power spectral **(B)** results in determining memory for list words and associative lures. In panel **(A)** behavioural results indicate a slight increase in accuracy in the recognition of associative lures across sleep and wake retention intervals. In panel **(B)** results indicate a complex relationship between theta power at encoding, memory type, and sleep/wake. Dashed line in **(A)** indicates chance performance.

Testing of hypothesis 1 indicated a significant interaction of theta power at encoding × condition × memory type interaction in producing memory outcomes, [χ^2^(1) = 43.81, *p* < 0.001], such that midline theta power increases at encoding resulted in the recognition of fewer list word memories for the wake condition but greater successful recognition for the sleep condition. This analysis demonstrated the opposite relationship for the endorsement of theme words (i.e., associations): relative theta decreases at encoding led to more endorsement of theme words and lower recognition of list words in the context of sleep (see Figure 4B).

**FIGURE 5:**
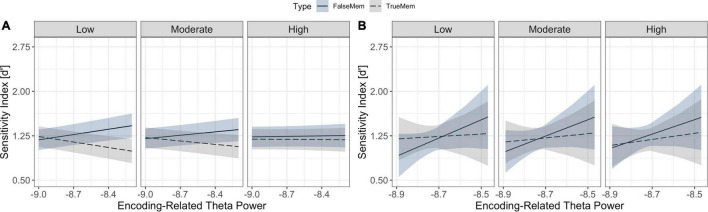
Relationships between sleep microstate variables and memory for list words and associative lures. **(A)** Interaction between theta power at encoding (x-axis), memory type (association in blue, veridical in grey), and sleep spindle density (facets) in predicting d’ (y-axis); **(B)** Interaction between theta power at encoding (x-axis), memory type (association in blue, veridical in grey), and sleep spindle-slow oscillation coupling strength (facets) in predicting d’ performance (y-axis). Ribbons indicate the 83% confidence interval.

Statistical analysis to test hypothesis 2 revealed a significant interaction between theta power and spindle density in determining memory for list words and associative lures, such that theta increases in conjunction with lower spindle density was associated with an increase in theme word recognition and a decrease in list word recognition [χ^2^(1) = 15.51, *p* < 0.001] (see Figure 5A). Similarly, we observed a significant interaction between slow oscillation-spindle coupling, memory type and theta power in determining outcomes [χ2(1) = 7.12, *p* = 0.008], such that theta power increases were differentially associated with memory for list words and lures across coupling strength levels (see Figure 5B). List word recognition was not modulated as a function of the interaction between slow oscillation-spindle coupling and theta power.

## Discussion

Here, we examined how encoding-related theta activity influences memory consolidation across sleep to facilitate learning and memory. We sought to test the idea that theta power at encoding would influence recognition of DRM list and theme words, potentially through the modulation of sleep-related oscillatory microstructure. To this end, we tested the relationship between encoding-related theta power and both sleep spindle density and slow oscillation-spindle coupling strength. Contrary to the first hypothesis (that relative theta power increases would lead to an increase in recognition of list words and a decrease in endorsement of associative theme words) we note an effect of midline theta decreases which differentially impacted memory between the wake and sleep conditions: wake resulted in improved recognition of list words and a decrease in endorsement of associative lures, both of which were tracked by encoding-related theta. Sleep was related to a decrease in recognition of list words and an increase in theme word endorsement, both linked to midline theta power decreases. This suggests that midline theta at encoding relates to both memory for experienced episodes and the association thereof and that this is differentially modulated by sleep and wake. As such, our results expand previous work on the role of encoding-related brain activity and how it may relate to sleep-based consolidation mechanisms.

Theta power during the encoding of information appears to play an important role in subsequent memory outcomes (Sederberg et al., 2003; Hasselmo and Stern, 2014). Our results indicate that theta power decreases at encoding differentially effects both recognition of previously seen, as well as gist memories, and has differential effects as a function of wake vs. sleep. These results are conceptually similar, but directionally different to those published in previous EEG studies, which have found that theta power increases, not decreases, relates to successful encoding of episodic memories (Kirov et al., 2009; Heib et al., 2015). This difference in findings can be explained by methodological differences between our study and previous literature. We have used a more direct measurement of theta power during encoding, whereas Heib et al. (2015) calculated the change in theta power from before to after a cued list word, and used this measure in their analyses. The cognitive implications of this difference may be that we have measured brain activity related to encoding a memory trace, whereas Heib et al. may have measured activity related to reconsolidation or other subsequent mnemonic activity. Similarly, our results focus around midline theta, whereas previous literature has noted an effect of frontal theta in general. Whether task or other non-specific functional differences relate to this discrepancy is an area for future research.

Previous literature (Heib et al., 2015; but more so Kirov et al., 2009) elegantly demonstrates that encoding theta power relates to memory outcomes across sleep. Typically, these findings are explained through an influence on hippocampocortical communication at theta frequency during wakeful encoding. Our study is unable to comment on whether this is specifically a “tagging” mechanism, as we did not include a manipulation to differentiate between memories to be consolidated and those to be ignored. Despite this, our results can still be interpreted through similar mechanisms as previously published, through the idea that theta may prime circuits for subsequent consolidation during sleep, and thus increase sleep spindle activity at these local sites during post-learning sleep (Petzka et al., 2021).

Our findings also demonstrate a clear relationship between encoding-related theta power and sleep spindle density. This partially supports the findings of Heib et al. (2015), with a difference of the directionality of theta power. These results further support the proposed effect of theta oscillations on consolidation through modulation of sleep spindle density (Heib et al., 2015). Encoding-related theta may potentially be an important marker of relevant encoding-related activity, which serves to prime circuits for subsequent consolidation, in much the same way that topographical overlap between encoding-related brain activity and sleep spindle density may relate to consolidation and memory outcomes across sleep (Petzka et al., 2021). Previous research can be updated based on this, mainly looking at a sleep spindle specific role in the consolidation of different types of memory (Fogel et al., 2007), such that gist processing is supported *via* higher spindle density along with theta power increases at encoding, and recognition of previously seen items showing the opposite trend. Sleep spindles have been implicated with integrating new information into existing knowledge (Tamminen et al., 2010), suggesting that their function reflects a more general process of learning as well as the hippocampocortical communication of encoded memory traces. It may be informative to consider the mechanisms through which encoding-related theta power and spindle density in subsequent sleep may lead to both improvements in memory, and the generalisation of encoded traces. Both mechanisms could be accounted for through statistical regularities in encoded information leading to greater replay for shared components over individual components, as is theorised in the competitive trace and IoTa accounts of memory (Lewis and Durrant, 2011; Yassa and Reagh, 2013). This idea has also been examined in memory literature to explain attributes of memory such as selective consolidation, item integration and associations, along with how consolidation binds that information together (Walker and Stickgold, 2010; Stickgold and Walker, 2013). A common mechanism to explain the capacity of the human brain to both use pattern separation processes to store veridical aspects of our experience, as well as to use pattern completion processes to extract meanings and rules therefrom would seem to be efficient. Our finding that different polarities of theta power at encoding may predict outcomes in terms of both memory and learning should be investigated more fully in future research, potentially through causal manipulations using associative memory paradigms, naps and non-invasive brain stimulation techniques, or different training modalities.

There are several limitations to the present study which should be considered in the interpretation of results. Considering that the DRM is fairly artificial, the generalisability of these findings should be tempered. Additionally, the DRM scores observed are higher than noted in other DRM studies (Pardilla-Delgado and Payne, 2017), which may be due to the shorter time frame between encoding and recognition, along with the effect of including more trials and an additional critical lure. Additional, nonspecific effects such as this may have influenced participants in their encoding and generalisation of information. This should not exert a marked influence on the EEG, although a more standard and controlled behavioural procedure would be of benefit in fortifying the present results. Further, considering that sleep loss prior to learning can negatively impact memory ability (Mander et al., 2008; Alberca-Reina et al., 2015), our manipulation of requiring participants to restrict their sleep by 1 h prior to encoding may have influenced their capacity to encode the material, although it is unlikely that any effects of this procedure were of a significant nature.

Future research could also focus on other mechanisms, in addition to the sleep-based oscillatory linkages we have described herein. For instance, modulations of cross-frequency coupling linked to measurable changes in task-related EEG could be another factor that could be investigated, as could how these metrics may be influenced by related sleep-based EEG phenomena. Research has also suggested that the aperiodic activity directly following a spindle may also be important for consolidation (Helfrich et al., 2021). The authors suggest the role of a sleep spindle is similar to that of being a messenger signal rather than the producer of consolidation itself, as is currently theorised. That is, by not analysing aperiodic activity around sleep spindles, we may have missed an important factor in sleep-based memory consolidation. This is a clear area of importance for future research.

With the idea that theta acts a tagging mechanism, the relationships found in the present study demonstrates that encoding-related theta activity can influence specific aspects of memory-related sleep neurophysiology, and highlights the connection between encoding and consolidation process in episodic memory. Consolidation is clearly impacted by processes tagged by theta power during encoding, and if models wish to develop a comprehensive view of memory consolidation, then extant theories of sleep-based memory consolidation should expand to include the neurobiological mechanisms underlying successful memory encoding.

## Data Availability Statement

The original contributions presented in the study are included in the article/Supplementary Material, further inquiries can be directed to the corresponding author.

## Ethics Statement

The studies involving human participants were reviewed and approved by University of South Australia Human Research Ethics. The patients/participants provided their written informed consent to participate in this study.

## Author Contributions

TG: data collection and manuscript preparation. ZC: data analysis and manuscript preparation. AC: study design, data analysis, and manuscript preparation. All authors contributed to the article and approved the submitted version.

## Conflict of Interest

The authors declare that the research was conducted in the absence of any commercial or financial relationships that could be construed as a potential conflict of interest.

## Publisher’s Note

All claims expressed in this article are solely those of the authors and do not necessarily represent those of their affiliated organizations, or those of the publisher, the editors and the reviewers. Any product that may be evaluated in this article, or claim that may be made by its manufacturer, is not guaranteed or endorsed by the publisher.
