# Supplementary material for: Theta Activity During Encoding Interacts With NREM Sleep Oscillations to Predict Memory Generalization
Source: Front Hum Neurosci. 2022 May 9;16:821191. doi: 10.3389/fnhum.2022.821191 (PMC9125147; doi:10.3389/fnhum.2022.821191)
Supplement: Supplementary file 1 [file Data_Sheet_1.docx]

Appendix 1: condition x type EMMs

|  | **DR** | | |
| --- | --- | --- | --- |
| *Predictors* | *Estimates* | *CI* | *p* |
| (Intercept) | 0.61 | 0.24 – 0.99 | **0.001** |
| theta | -0.00 | -0.07 – 0.06 | 0.965 |
| Condition[S] | 0.04 | -0.23 – 0.32 | 0.767 |
| type[FalseMem] | 0.17 | -0.01 – 0.35 | 0.063 |
| prestim | 0.00 | -0.08 – 0.08 | 0.956 |
| IR | 0.43 | 0.40 – 0.45 | **<0.001** |
| theta * Condition[S] | 0.00 | -0.03 – 0.03 | 0.991 |
| theta * type[FalseMem] | 0.01 | -0.01 – 0.03 | 0.231 |
| Condition[S] * type[FalseMem] | -0.61 | -0.79 – -0.44 | **<0.001** |
| theta * Condition[S] * type[FalseMem] | -0.07 | -0.09 – -0.05 | **<0.001** |
| **Random Effects** | | | |
| σ^2^ | 0.02 | | |
| τ_00_ _Channel_ | 0.00 | | |
| τ_00_ _subj_ | 0.13 | | |
| N _subj_ | 35 | | |
| N _Channel_ | 56 | | |
| Observations | 2456 | | |
| Marginal R^2^ / Conditional R^2^ | 0.654 / NA | | |

Appendix 2: condition x laterality x type EMMs

|  | **DR** | | |
| --- | --- | --- | --- |
| *Predictors* | *Estimates* | *CI* | *p* |
| (Intercept) | 0.51 | -3.43 – 4.44 | 0.800 |
| theta | -0.00 | -0.43 – 0.42 | 0.990 |
| Density | 0.03 | -1.01 – 1.08 | 0.949 |
| type[FalseMem] | 10.62 | 8.70 – 12.55 | **<0.001** |
| sag[anterior] | 0.01 | -2.98 – 2.99 | 0.997 |
| sag[central] | -0.01 | -3.13 – 3.11 | 0.997 |
| lat[Left] | 0.00 | -2.83 – 2.84 | 0.999 |
| lat[midline] | 0.01 | -2.55 – 2.56 | 0.997 |
| IR | 0.44 | 0.38 – 0.51 | **<0.001** |
| prestim | -0.01 | -0.35 – 0.34 | 0.965 |
| theta * Density | 0.00 | -0.12 – 0.12 | 0.952 |
| theta * type[FalseMem] | 1.22 | 1.00 – 1.44 | **<0.001** |
| Density * type[FalseMem] | -2.71 | -3.36 – -2.06 | **<0.001** |
| theta * sag[anterior] | 0.00 | -0.34 – 0.35 | 0.998 |
| theta * sag[central] | -0.00 | -0.36 – 0.36 | 0.998 |
| Density * sag[anterior] | -0.01 | -1.10 – 1.07 | 0.982 |
| Density * sag[central] | 0.01 | -1.03 – 1.04 | 0.991 |
| type[FalseMem] * sag[anterior] | 0.20 | -2.44 – 2.85 | 0.880 |
| type[FalseMem] * sag[central] | 2.71 | -0.23 – 5.65 | 0.070 |
| theta * lat[Left] | 0.00 | -0.32 – 0.33 | 0.999 |
| theta * lat[midline] | 0.00 | -0.29 – 0.30 | 0.997 |
| Density * lat[Left] | -0.00 | -0.92 – 0.92 | 0.996 |
| Density * lat[midline] | -0.00 | -0.89 – 0.88 | 0.994 |
| type[FalseMem] * lat[Left] | 0.62 | -2.20 – 3.43 | 0.667 |
| type[FalseMem] * lat[midline] | -2.30 | -4.80 – 0.21 | 0.072 |
| sag[anterior] * lat[Left] | -0.01 | -3.85 – 3.82 | 0.995 |
| sag[central] * lat[Left] | 0.01 | -4.39 – 4.41 | 0.996 |
| sag[anterior] * lat[midline] | -0.03 | -3.74 – 3.69 | 0.988 |
| sag[central] * lat[midline] | -0.01 | -3.92 – 3.89 | 0.995 |
| theta * Density * type[FalseMem] | -0.31 | -0.39 – -0.24 | **<0.001** |
| theta * Density * sag[anterior] | -0.00 | -0.13 – 0.12 | 0.983 |
| theta * Density * sag[central] | 0.00 | -0.12 – 0.12 | 0.991 |
| theta * type[FalseMem] * sag[anterior] | 0.04 | -0.27 – 0.35 | 0.795 |
| theta * type[FalseMem] * sag[central] | 0.31 | -0.03 – 0.65 | 0.071 |
| Density * type[FalseMem] * sag[anterior] | -0.49 | -1.42 – 0.44 | 0.300 |
| Density * type[FalseMem] * sag[central] | -0.51 | -1.48 – 0.47 | 0.305 |
| theta * Density * lat[Left] | -0.00 | -0.11 – 0.10 | 0.996 |
| theta * Density * lat[midline] | -0.00 | -0.10 – 0.10 | 0.995 |
| theta * type[FalseMem] * lat[Left] | 0.07 | -0.26 – 0.39 | 0.680 |
| theta * type[FalseMem] * lat[midline] | -0.26 | -0.55 – 0.03 | 0.081 |
| Density * type[FalseMem] * lat[Left] | -0.09 | -1.01 – 0.82 | 0.839 |
| Density * type[FalseMem] * lat[midline] | 0.55 | -0.32 – 1.43 | 0.213 |
| theta * sag[anterior] * lat[Left] | -0.00 | -0.44 – 0.44 | 0.995 |
| theta * sag[central] * lat[Left] | 0.00 | -0.50 – 0.51 | 0.996 |
| theta * sag[anterior] * lat[midline] | -0.00 | -0.43 – 0.43 | 0.989 |
| theta * sag[central] *lat[midline] | -0.00 | -0.45 – 0.45 | 0.995 |
| Density * sag[anterior] * lat[Left] | 0.00 | -1.29 – 1.29 | 0.995 |
| Density * sag[central] * lat[Left] | -0.01 | -1.41 – 1.39 | 0.991 |
| Density * sag[anterior] * lat[midline] | 0.02 | -1.33 – 1.36 | 0.982 |
| Density * sag[central] * lat[midline] | 0.01 | -1.32 – 1.34 | 0.989 |
| type[FalseMem] * sag[anterior] * lat[Left] | -0.88 | -4.70 – 2.94 | 0.651 |
| type[FalseMem] * sag[central] * lat[Left] | 0.81 | -3.57 – 5.19 | 0.717 |
| type[FalseMem] * sag[anterior] * lat[midline] | 2.45 | -1.17 – 6.07 | 0.184 |
| type[FalseMem] * sag[central] * lat[midline] | -0.70 | -4.57 – 3.17 | 0.722 |
| theta * Density * type[FalseMem] * sag[anterior] | -0.06 | -0.17 – 0.05 | 0.265 |
| theta * Density * type[FalseMem] * sag[central] | -0.06 | -0.17 – 0.05 | 0.307 |
| theta * Density * type[FalseMem] * lat[Left] | -0.01 | -0.11 – 0.09 | 0.851 |
| theta * Density * type[FalseMem] * lat[midline] | 0.06 | -0.04 – 0.16 | 0.226 |
| theta * Density * sag[anterior] * lat[Left] | 0.00 | -0.15 – 0.15 | 0.995 |
| theta * Density * sag[central] * lat[Left] | -0.00 | -0.16 – 0.16 | 0.991 |
| theta * Density * sag[anterior] * lat[midline] | 0.00 | -0.15 – 0.16 | 0.983 |
| theta * Density *sag[central] * lat[midline] | 0.00 | -0.15 – 0.15 | 0.989 |
| theta * type[FalseMem] * sag[anterior] * lat[Left] | -0.10 | -0.54 – 0.34 | 0.650 |
| theta * type[FalseMem] * sag[central] * lat[Left] | 0.09 | -0.41 – 0.59 | 0.728 |
| theta * type[FalseMem] * sag[anterior] * lat[midline] | 0.28 | -0.14 – 0.70 | 0.188 |
| theta * type[FalseMem] * sag[central] * lat[midline] | -0.07 | -0.52 – 0.37 | 0.744 |
| Density * type[FalseMem] * sag[anterior] * lat[Left] | 0.30 | -0.99 – 1.58 | 0.651 |
| Density * type[FalseMem] * sag[central] * lat[Left] | -0.38 | -1.77 – 1.02 | 0.594 |
| Density * type[FalseMem] * sag[anterior] * lat[midline] | -0.70 | -2.01 – 0.60 | 0.290 |
| Density * type[FalseMem] * sag[central] * lat[midline] | 0.52 | -0.80 – 1.84 | 0.441 |
| theta * Density * type[FalseMem] * sag[anterior] * lat[Left] | 0.03 | -0.11 – 0.18 | 0.648 |
| theta * Density * type[FalseMem] * sag[central] * lat[Left] | -0.04 | -0.20 – 0.12 | 0.601 |
| theta * Density * type[FalseMem] * sag[anterior] * lat[midline] | -0.08 | -0.23 – 0.07 | 0.291 |
| theta * Density * type[FalseMem] * sag[central] * lat[midline] | 0.06 | -0.09 – 0.21 | 0.455 |
| **Random Effects** | | | |
| σ^2^ | 0.02 | | |
| τ_00_ _subj_ | 0.20 | | |
| τ_00_ _Channel_ | 0.00 | | |
| N _subj_ | 13 | | |
| N _Channel_ | 11 | | |
| Observations | 286 | | |
| Marginal R^2^ / Conditional R^2^ | 0.838 / NA | | |
